# Surgical management of tympanojugular paragangliomas using the flexible CO_2_ laser

**DOI:** 10.1007/s00405-022-07416-5

**Published:** 2022-05-05

**Authors:** Stephan Hackenberg, Till Jasper Meyer, Johannes Häfner, Matthias Scheich, Manuel Stöth, Fadi Al-Tinawi, Tilmann Neun, Robert Mlynski, Rudolf Hagen, Agmal Scherzad

**Affiliations:** 1 Department of Otorhinolaryngology - Head and Neck Surgery, RWTH Aachen University Hospital, Aachen, Germany; 2 Department of Oto-Rhino-Laryngology, Plastic, Aesthetic and Reconstructive Head and Neck Surgery, Würzburg University Hospital, Würzburg, Germany; 3 Institute for Diagnostic and Interventional Neuroradiology, Würzburg University Hospital, Würzburg, Germany; 4 Department of Otorhinolaryngology, Head and Neck Surgery “Otto Körner”, Rostock University Medical Center, Rostock, Germany

**Keywords:** Tympanojugular paraganglioma, Tympanic paraganglioma, Jugular paraganglioma, Surgical management of paraganglioma, Laser surgery, Flexible CO_2_ laser

## Abstract

**Purpose:**

Surgery is a standard therapy for tympanojugular paragangliomas (TJP). Maintaining the quality of life (QoL) requires functional preservation. The flexible CO_2_ laser allows contact-free tumor removal. This retrospective study compares the postoperative functional outcomes of TJP surgery with and without the flexible CO_2_ laser.

**Methods:**

Between 2005 and 2019, 51 patients with TJP were surgically treated at a tertiary hospital. Until 2012, 17 patients received conventional surgery. Thereafter, the flexible laser was used in 34 patients. Tumor extend, pre- and postoperative cranial nerve function, and complications were compared between the groups.

**Results:**

The cohort consisted of 33 class A and B tumors and 18 class C and D tumors. Preoperative embolization was performed in 17 cases. Class C/D TJP were usually removed via an infratemporal fossa type A approach. Gross total tumor removal was achieved in 14/18 class C/D tumors. 3/51 patients suffered from long-term partial or complete facial palsy. No differences in post-therapeutic cranial nerve function or complications were noted between the conventional and laser group. One recurrence was observed after complete tumor resection.

**Conclusion:**

The flexible CO_2_ laser was shown to be a safe and effective alternative to conventional bipolar cauterization, which is appreciated by the surgeon in these highly vascularized tumors. Both techniques allowed a high tumor control rate and good long-term results also from a functional point of view.

## Introduction

Paragangliomas are tumors arising from the paraganglial cells, secretory active neuroendocrine cells located in several anatomical regions of the human body. The subgroup of tympanojugular paragangliomas (TJP), formally known as glomus jugulare and tympanicum tumors, originate from paraganglia cells in the adventitia of the jugular bulb [1]. TJP are benign skull base lesions. However, they present as locally destructive and aggressively extending tumors. They erode the bone of the middle ear and variable parts of the temporal bone, may invade the upper neck, and infiltrate the dura with intracranial extension into the middle or posterior fossa. In 1988, Ugo Fisch published a widely accepted classification for TJP, categorizing the tumors according to their extent based on radiologic criteria (Table 1) [2].

**Table 1:** Classification of tympanojugular paragangliomas [according to Fisch and Mattox, 1988]

Class A	Tumor limited to the middle ear
Class B	Tumor limited to the tympanomastoid area with no infralabyrinthine compartment involvement
Class C	Tumor involving the infralabyrinthine compartment of the temporal bone and extending into the petrous apex
Class C1	Limited involvement of the vertical portion of the carotid canal
Class C2	Invasion of the vertical portion of the internal carotid artery canal
Class C3	Invasion of the horizontal portion of the internal carotid artery canal
Class C4	Invasion of the foramen lacerum and the cavernous sinus
Class D	Tumor with an intracranial extension
Class De	Extradural extension
Class Di	Intradural extension
Class D1	Smaller than 2 cm in diameter
Class D2	Larger than 2 cm in diameter

Radiological diagnostics of TJP include high-resolution contrast-MRI of the skull base and CT-scan of the temporal bone. TJP are hyperintense in the T2-weighted MRI. After contrast agent administration, the strong vascularization leads to a strong enhancement. Digital subtraction angiography (DSA) allows the detection of even small tumors [3]. Large TJP (class C and D) can be subdivided according to the involvement of the internal carotid artery or intracranial extension. There are several options for the management of TJP that need to be discussed in multi-disciplinary teams of experts in head and neck and skull base surgery. Therapeutic strategies are determined individually based on specific patient-related parameters, tumor size, and genetic profile [4]. The therapy of TJP has been controversially discussed for decades. Although fractionated radiotherapy, stereotactic radiotherapy, gamma knife and cyberknife radiosurgery are accepted treatment options, surgery is considered to be the main therapeutic approach by many authors [5]. In large TJP, preoperative angiography and embolization with, e.g., polyvinyl alcohol particles are recommended. However, surgical therapy of class C and D TJP is challenging due to the high vascularization of the lesions and their anatomical relationship to the internal carotid artery, cranial nerves and dura [6]. Quality of life mainly depends on the preservation or functional reconstruction of these structures. The facial nerve has an outstanding importance in the surgical treatment of TJP. The mastoid subsegment of the facial nerve lies on the surgical pathway to the jugular bulb. Thus, appropriate management of the facial nerve is critical in the surgical treatment of class C/D TJP. Since in most cases, the tumor only erodes the bony canal of the nerve without infiltrating the epineurium, a classical nerve rerouting as an obligatory part of the infratemporal fossa type A approach is appropriate [7]. Nerve resections are only necessary in cases of infiltration [8].

The flexible CO_2_ laser fiber was introduced in 2002. The possibility to guide the laser beam through straight or curved applicators allows precise and targeted manipulations of complex anatomical sites, which made this technology attractive for several microsurgical approaches [9, 10]. Since 2010, flexible CO_2_ laser fiber supplements conventional bipolar cauterization techniques in vestibular schwannoma and TJP surgery at the study center [10]. The aim of this retrospective study was to assess the functional outcome and tumor control in surgically treated TJP, comparing flexible CO_2_ laser surgery with conventional surgery.

## Materials and methods

### Patient data

Patients who had undergone surgery for previously untreated TJP at a tertiary hospital from January 2006 to February 2019 were retrospectively reviewed for clinical data. Patients requiring revision surgery were excluded. During the observed period, 61 patients had surgery for paraganglioma of the head and neck. Glomus vagale (*n* = 1) and glomus caroticum tumors (*n* = 9) were excluded. Hence, 51 patients with TJP class A, B, C and D were finally identified. Perioperative and follow-up data were analyzed using patients’ charts medical records. The minimum and maximum follow-up periods were 1 year and 10 years, respectively. In 17 cases, the tumor was removed by conventional bipolar cauterization; in 34 cases, the flexible CO_2_ laser fiber was applied. The surgeries were performed by the same group of surgeons (R.H., R.M., S.H.) in both groups, respectively. Each patient underwent a comprehensive radiological examination with high-resolution CT-scan of the temporal bone and contrasted magnet resonance tomography (MRI) of the skull base. Furthermore, angiographic evaluation was carried out in patients with class C and D tumors (digital subtraction angiography, DSA). Diagnostic and interventional radiological procedures were performed by a highly specialized neuroradiological team. Each patient received pure-tone audiometry once prior to surgery and during follow-up. Audiologic results were summarized in pure-tone averages (PTA) of the air conduction and bone conduction thresholds of the frequencies 500 Hz, 1,000 Hz, 2,000 Hz and 4,000 Hz. For audiological analysis, only patients with existing pre- and post-operative audiological data were included. The facial and lower cranial nerve function were monitored prior to treatment and during follow-up. Facial nerve function was assessed using the House-Brackmann classification [11].

### Surgery

Intraoperative electrophysiological monitoring, including facial nerve (Neurosign 100, Magstim Co Ltd. Wales, UK) was applied in every TJP procedure. The surgical approach depended on the extent of the tumor. While small and localized tumors (class A, B, partially C) were resected transmeatally with tympanoplastic reconstruction using a retroauricular approach, large tumors with extended manifestations at the jugular foramen, the internal carotid artery, or infiltration of the dura (class C and D) required to access via the infratemporal fossa type A approach. Furthermore, neuroradiological interventional angiography was performed two days prior to surgery of class C and D tumors. The infratemporal fossa type A approach involved preoperative super-selective embolization of the tumor feeder vessels using polyvinyl alcohol particles. Angiography and embolization were not necessary for small tumors (class A and B), which were dissected transmeatally and transtympanally using a retroauricular approach. Retroauricular tumor removal was followed by immediate tympanoplastic and ossicular reconstruction (if necessary) during the same procedure. The initial steps of the infratemporal fossa type A approach were neck dissection, identification of the facial nerve at the stylomastoid foramen, identification of the cranial nerves X, XI, XII as well as the internal carotid artery; and ligation of the internal jugular vein. Then, an extended mastoidectomy, including exposure and closure of the sigmoid sinus, was then performed. The middle ear and the external auditory canal were removed completely, the stapes were preserved. The vertical part of the internal carotid artery was exposed in all cases. After a wide exposure of the dura of the posterior and the middle fossa, the facial nerve was exposed and skeletonized from the horizontal semicircular canal to the stylomastoid foramen. Usually, small feeder vessels from the adventitia of the internal carotid artery and the perineurium of the facial nerve had to be coagulated carefully. To reach the tumor manifestations at the jugular bulb, the nerve was transposed anteriorly, the so-called “anterior rerouting”. Tumor shrinkage was performed either by bipolar coagulation or by the flexible CO_2_ laser fiber (Omniguide, FELS 300A, Cambridge, MA, USA). The tumor was vaporized using 5 W in a continuous wave mode and 70 psi pressure of helium gas for cooling the flexible fiber. If affected, dura to the posterior fossa was resected. In cases of tumor invasion of the medial wall of the jugular bulb, special care had to be taken to preserve the lower cranial nerves. A complete removal of the tumor was attempted in all cases. The identification of the lower cranial nerves was especially difficult in large TJP. At the end of the surgery, bleeding from the inferior petrosal sinus was stopped by Tabotamp (Ethicon, Raritan, New Jersey, USA) packing. Dural reconstruction was performed with TachoSil (Takeda, Tokyo, Japan). Specimens for histopathological and genetic examination were preserved. Finally, the petrosectomy cavity was filled with an absorbable gelatin sponge (Gelita Medical, Eberbach, Germany) and obliteration of the external auditory canal was performed. Surgery time was defined as the period from skin incision and closure.

## Results

### Baseline data

A total of 51 TJP were included in our study from 2005 to 2019. The mean age of the patients was 58 years, ranging from 23 to 79 years. Female patients were more often affected with a sex distribution (female to male) of 42 to 9. Tumor extends according to the Fisch classification and the surgical approach are summarized in Table 2.

**Table 2:** Summary of tumor extend and surgical approach

Fisch classification	Total *n*	Retroauricular approach *n* (%)	Infratemporal fossa type A approach *n* (%)
A	9	9 (100%)	0
B	24	22 (92%)	2 (8%)
C1 D0	8	1 (13%)	7 (87%)
C1 De2 Di1	1	0	1 (100%)
C2 D0	1	0	1 (100%)
C2 De1	4	0	4 (100%)
C2 De2	2	0	2 (100%)
C2 De1 Di1	2	0	2 (100%)
C3 D0	0	0	0
Total	51	32 (63%)	19 (37%)

Preoperative symptoms of patients with a TJP class A and B included pulse-synchronous tinnitus (24/33), fullness in the ear (3/33), and hearing loss (19/33). Patients with TJP class C and D mainly reported about pulse-synchronous tinnitus (14/18), hearing loss (8/18), fullness in the ear (7/18), vertigo or dizziness (4/18), dysphonia (3/18), and dysphagia (1/18). Preoperatively, angiography with embolization was performed in 17 out of 19 patients who underwent the infratemporal fossa type A approach later. The two other patients only received angiography but without embolization due to an inconclusive feeder vessel architecture. Typical feeder vessels were the ascending pharyngeal artery (15), occipital artery (5), basilar artery (1), and superficial temporal artery (1). In three cases, a second neuroradiological intervention was necessary due to insufficient embolization after the first session.

Resection of the tumor with the flexible CO_2_ laser was performed in 34 patients, while 17 tumors were operated conventionally. Both surgical methods (laser and conventional) were applied in retroauricular as well as infratemporal approaches, respectively. Surgical methods and approaches are summarized in Table 3. The follow-up period was between 1 to 10.2 years.

**Table 3:** Summary of surgical method and surgical approach

	Total *n*	Retroauricular approach	Infratemporal fossa type A approach
Conventional surgery	17	9 (53%)	8 (47%)
Flexible CO_2_ laser surgery	34	23 (68%)	11 (32%)
Total	51	32 (63%)	19 (37%)

### Cranial nerve function

No cranial nerve lesions were observed after TJP surgery by the retroauricular approach (9 class A tumors, 22 class B tumors, one class C1 tumor, *n* = 32).

Thus, the following chapter focusses on the patients who had received surgery via the infratemporal fossa type A approach (*n* = 19). Eight patients were operated conventionally and 11 patients with the flexible CO_2_ laser. Table 4 shows a comparison of pre- and postoperative cranial nerve function in TJP patients via the infratemporal fossa type A approach.

**Table 4:** Postoperative facial nerve (VII) and lower cranial nerve (LCN) function

Facial nerve (VII) function within the first postoperative period (< 6 months after surgery)				
	Conventional surgery (*n* = 8)		Laser surgery (*n* = 11)	
	VII intact *n* (%)	VII palsy *n* (%)	VII intact *n* (%)	VII palsy *n* (%)
Class B	1 (100%)	0	1 (100%)	0
Class C	1 (20%)	4 (80%)	1 (33%)	2 (67%)
Class D	1 (50%)	1 (50%)	5 (71%)	2 (29%)

Facial nerve (VII) function in long-term follow-up (> 6 months after surgery), one case lost to follow-up				
	Conventional surgery (*n* = 8)		Laser surgery (*n* = 10)	
	VII intact *n* (%)	VII palsy *n* (%)	VII intact *n* (%)	VII palsy *n* (%)
Class B	1 (100%)	0	1 (100%)	0
Class C	5 (100%)	0	2 (67%)	1 (33%)
Class D	1 (50%)	1 (50%)	5 (83%)	1 (17%)

Lower cranial nerve (LCN) function in long-term follow up				
	Conventional surgery (*n* = 8)		Laser surgery (*n* = 11)	
	LCN intact *n* (%)	LCN impairment *n* (%)	LCN intact *n* (%)	LCN impairment *n* (%)
Class B	1 (100%)	0	1 (100%)	0
Class C	3 (60%)	2 (40%)	1 (33%)	2 (67%)
Class D	1 (50%)	1 (50%)	5 (71%)	2 (29%)

The most common postoperative complication was a temporary facial nerve palsy, which occurred in 9 out of 19 patients (47%). Five palsies were observed in the subgroup after conventional surgery (total *n* = 8) and four paralyses were observed in the subgroup after flexible CO_2_ laser surgery (total *n* = 11). No preoperative facial nerve palsy was seen in our total cohort. Facial nerve resection was not necessary in any case since there was no patient with nerve infiltration by the tumor. Regarding long-term follow-up, facial nerve function recovered completely in five cases, one patient showed a partial palsy (House-Brackmann III), and two patients remained with a complete paralysis (one patient out of the conventional and one out of the laser subgroup, respectively). One patient discontinued follow-up. No iatrogenic sensorineural hearing loss was detected in our cohort. Regarding the lower cranial nerves, one patient presented with preoperative inability to elevate the arm above the horizontal without externally rotating, indicating paralysis of the accessory nerve. Three patients presented a nerve XI palsy after treatment, two within the conventionally resected subgroup and one in the subgroup after flexible CO_2_ laser surgery. Two patients suffered from vagal nerve palsy preoperatively, which did not improve after surgery. Two additional patients with regular preoperative vagal nerve function developed a palsy after surgery. Specifying the four patients with vagal nerve palsy, two were in the conventional and two in the laser group. Additionally, one patient presented a preoperative glossopharyngeal nerve palsy, which did not improve after surgery. This patient had a vagal nerve palsy as well. There was no patient with an isolated palsy of the glossopharyngeal nerve. Fortunately, no case of severe dysphagia or aspiration occurred, so the placement of a PEG tube was not necessary. Oral nutrition was sufficiently possible in all patients.

### Surgery time, intraoperative blood loss and tumor resection status

All tumors could be removed by single-stage resection. No differences were observable regarding surgery time between the conventional group and the flexible CO_2_ laser group. Mean surgery time for the retroauricular approach was 129 min in both groups. Regarding the infratemporal fossa type A approach, the mean surgery time was 386 min in the conventional cohort and 410 min in the laser cohort. Red blood cells (RBCs) transfusion were necessary for four patients during infratemporal fossa type A approach surgery. One patient received one RBCs concentrate unit, two patients received two units each, and one patient received four units. The definition of gross total tumor resection was based on the surgeon’s report and if no residual tumor was seen in the 12-months follow-up MRI scan. Partial/subtotal resections were performed in five out of 19 cases of infratemporal fossa type A surgery. Gross total tumor resections were possible in 14 patients. In the retroauricular subgroup, one out of 32 patients received subtotal resection (Fisch C1 D0), while the rest of the patients within this group could be operated completely. The most common reason for subtotal tumor resection was its proximity to the functional intact lower cranial nerves and internal carotid artery. One recurrence was seen after 7 years in the patient group with gross total tumor resection. This patient was in the laser group.

### Audiological results

**Fig. 1:**
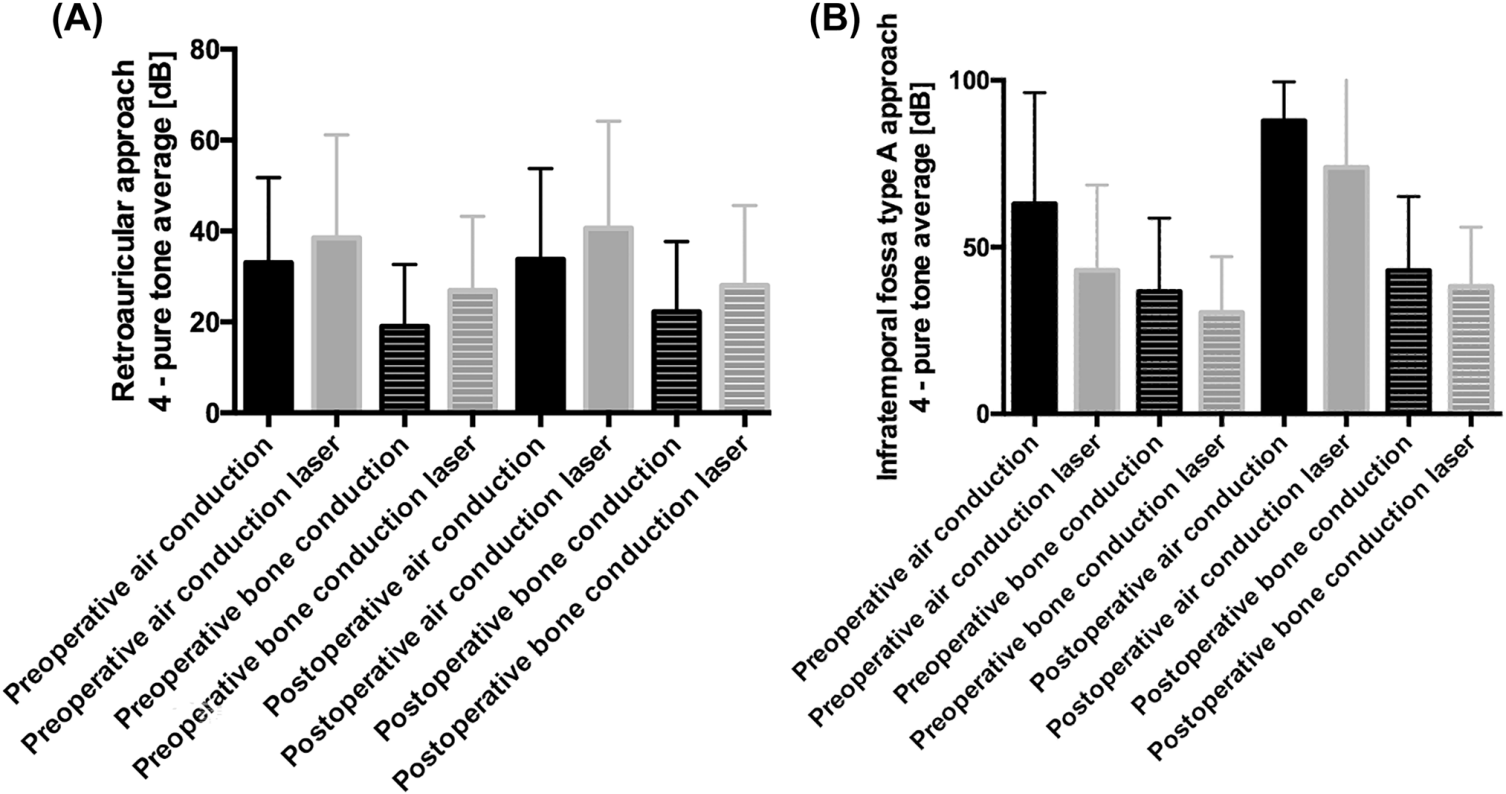
Pre- and post-operative audiology results in the **A** retroauricular approach and **B** infratemporal fossa type A approach. **A** There is no relevant difference between the pre- and post-operative audiology findings in the conventional (*n* = 9) and laser (*n* = 16) surgery group after retroauricular approach. **B** There is an increase in the postoperative air conduction in the conventional (*n* = 3) and laser (*n* = 9) surgery group after infratemporal fossa type A approach

The pre- and postoperative audiological findings in the conventional (*n* = 9) and laser (*n* = 16) surgery group after retroauricular approach show no relevant differences (see Fig. 1). After the infratemporal fossa type A approach, the postoperative air–bone gap increases both, in the conventional (*n* = 3) and laser (*n* = 9) surgery group (see Fig. 1), resulting in a complete loss of air conduction. No significant postoperative decrease in bone conduction was seen in our cohort.

## Discussion

TJP are rare, slowly progressive and highly vascularized tumors. The growth pattern is diffuse and due to the close anatomical relationship to important structures, there is a frequent involvement of nerves and vessels. These structures include, for example, the internal carotid artery and the facial nerve as well as the lower cranial nerves. Nerve impairment can result in a variety of clinical symptoms associated with a significant reduction of quality of life. Clinically, TJP is most notable for pulse-synchronous tinnitus and cranial nerve palsies. Diagnostic approaches must include clinical examination, audiometry, assessment of cranial nerve function, CT-scan and MRI, nuclear medical evaluation by ^68^GaDOTA-TOC PET scan and genetic testing. Therapeutic strategies are controversially discussed. However, surgical resection is accepted as the treatment of choice by many authors, although it also carries the risk of intraoperative and postoperative complications [12]. Since the publication of Fisch’s classification, the surgical approach has not changed significantly. Different modifications were made to minimize the above-mentioned intra- and postoperative complications and to improve the surgical outcome depending on the tumor extent [13]. Decision-making in the management of TJP is complex and should involve a multi-disciplinary team. While in most patients, there is an indication for treatment, watchful waiting may be an option in very few cases. According to Smith et al., these include very small and asymptomatic tumors, co-morbidities and advanced age of the patients as well as SDHx mutations indicating a low malignancy risk [4]. In contrast to this statement, we believe that patients with small tumors should receive surgery in the first instance because functional deficits are very rare and a general eligibility for surgery is assumed. Our data support this opinion.

The application of the CO_2_ laser is an established procedure in otorhinolaryngology. Due to the physical properties of the laser beam, which can only be directed in a straight way, the application in complex anatomical regions like the skull base is limited. Consequently, flexible CO_2_ laser fibers seem to be a possible alternative in frontal and lateral skull base surgery. At our department, the flexible CO_2_ laser is routinely applied in microsurgery of vestibular schwannoma via the middle cranial fossa approach or the translabyrinthe approach. A study of our own group could demonstrate equivalent results in using the flexible CO_2_ laser compared to bipolar tumor cauterization [10]. Durvasula and colleagues performed laser surgical resection in nine patients with TJP class A and B [14]. The quintessence of the study was a reduction of bleeding as well as excellent tumor control by a diode or KTP laser surgery. In the present study, both small class A/B tumors and extended class C/D tumors were operated on using the conventional bipolar coagulation technique as well as the flexible CO_2_ laser. We did not find any striking functional outcome differences between conventional surgery and the use of the flexible CO_2_ laser. Due to our small cohort and the limited number of events, statistical analysis of the results was not reasonable. Instead, results were described in detail in order to allow interpretation. Regarding surgery time, preservation of nerve function and management of hemostasis, the use of the flexible CO_2_ laser compared with bipolar cauterization seems to be non-inferior. A comparison of the functional outcome results in the present study to results of other groups in the literature with similar cohorts reveals coequal findings [12, 15]. The risk for cranial nerve injury or incomplete tumor resection is directly related to the tumor extend. The most affected structure is the facial nerve. Jansen et al. reported on excellent outcome in class A and B tumors, while patients with class C and D tumors develop significant post-therapeutic impairments [16]. Mario Sanna and his group published multiple papers on the management of TJP covering an impressive and unique cohort including over 230 patients [17]. Publications of this group focus on very special techniques and subgroups like extended surgical approaches [7], vertebral artery involvement [18], intradural extension [19] or revision surgery [20]. Their results are not comparable to the data of other groups. Often, functional outcome is worse, compared to unselected cohorts, due to the complexity of the reported cases. Gross total tumor removal should generally be achieved in TJP surgery. However, this aim is not attainable in all cases to prevent surgery-related neural or brain damage. Mazzoni and Zanoletti demonstrated that partial resection and observation may be an option for TJP therapy in carefully selected cases of class C and D findings [21]. In our cohort, five class C TJP were not removed completely to prevent vagal nerve damage or internal carotid artery injury. Subtotal resection was either found in the conventional and in the laser subgroup. Our partial surgery cases are intensively controlled by MRI and in none of the cases, revision surgery was necessary due to relevant growth of the residual tumor or newly installed nerve paralysis since now.

As reported before, TJP patients characteristically show a combined hearing loss [14, 22, 23]. Comparing the functional results according to the audiology findings, there was no relevant difference between the conventional and laser surgery group after retroauricular and infratemporal fossa type A approach. Due to the surgical technique, the infratemporal fossa type A approach is associated with a complete postoperative air–bone gap. Both in the conventional and in the laser group, no sensorineural hearing deterioration was seen after surgery.

Financial aspects play a certain role in deciding whether to use the flexible CO_2_ laser versus the ring forceps bipolar cauterization technique. The laser fiber usually is available as a single-use device with an approximated cost of 700–1000 €. Thus, additional costs occur and have to be taken into account. Since functional outcome and surgery time is not superior in laser surgery patients, the relevant benefit of the laser system is comfort for the surgeon. Due to the continuous gas flow during laser application, the sight onto the surgical field is good and facilitates subtle preparation.

One limitation of the study is the retrospective design and therefore the missing randomization of the therapeutic approach.

In summary, functional outcome and tumor control were equivalent in both cohorts, the laser and the conventional group. The flexible CO_2_ laser proved to be non-inferior to the bipolar technique. Thus, tumor vaporization with the laser is a safe procedure in TJP surgery. Furthermore, contact-free coagulation and removal of blood by the laser-associated helium gas stream are comfortable for the surgeon. On the other hand, access to a flexible CO_2_ laser and appropriate equipment is costly and not available in all clinics.

## Conclusion

The use of the flexible CO_2_ laser fiber can be an alternative method to conventional bipolar cauterization in TJP tumor surgery.

## Data Availability

All data are available on request from the corresponding author.
